# Comparative Evaluation of Sloppy Molecular Beacon and Dual-Labeled Probe Melting Temperature Assays to Identify Mutations in *Mycobacterium tuberculosis* Resulting in Rifampin, Fluoroquinolone and Aminoglycoside Resistance

**DOI:** 10.1371/journal.pone.0126257

**Published:** 2015-05-04

**Authors:** Sandy S. Roh, Laura E. Smith, Jong Seok Lee, Laura E. Via, Clifton E. Barry, David Alland, Soumitesh Chakravorty

**Affiliations:** 1 Department of Medicine, New Jersey Medical School, Rutgers University, Newark, New Jersey, United States of America; 2 Department of Microbiology, International Tuberculosis Research Center, Changwon, Gyeongsang, Republic of Korea; 3 Tuberculosis Research Section, LCID, NIAID, NIH, Bethesda, MD, United States of America; University of Padova, Medical School, ITALY

## Abstract

Several molecular assays to detect resistance to Rifampin, the Fluoroquinolones, and Aminoglycosides in *Mycobacterium tuberculosis* (*M*. *tuberculosis*) have been recently described. A systematic approach for comparing these assays in the laboratory is needed in order to determine the relative advantage of each assay and to decide which ones should be advanced to evaluation. We performed an analytic comparison of a Sloppy Molecular Beacon (SMB) melting temperature (Tm) assay and a Dual labeled probe (DLP) Tm assay. Both assays targeted the *M*. *tuberculosis rpoB*, *gyrA*, *rrs* genes and the *eis* promoter region. The sensitivity and specificity to detect mutations, analytic limit of detection (LOD) and the detection of heteroresistance were tested using a panel of 56 clinical DNA samples from drug resistant *M*. *tuberculosis* strains. Both SMB and DLP assays detected 29/29 (100%) samples with *rpoB* RRDR mutations and 3/3 (100%) samples with *eis* promoter mutations correctly. The SMB assay detected all 17/17 *gyrA* mutants and 22/22 *rrs* mutants, while the DLP assay detected 16/17 (94%) *gyrA* mutants and 12/22 (55%) *rrs* mutants. Both assays showed comparable LODs for detecting *rpoB* and *eis* mutations; however, the SMB assay LODs were at least two logs better for detecting wild type and mutants in *gyrA* and *rrs* targets. The SMB assay was also moderately better at detecting heteroresistance. In summary, both assays appeared to be promising methods to detect drug resistance associated mutations in *M*. *tuberculosis*; however, the relative advantage of each assay varied under each test condition.

## Introduction

Tuberculosis (TB) is an increasing health concern worldwide. Despite overall reduction in the rate of new cases of TB, increased incidence of multidrug resistant (MDR) and extensively drug resistant (XDR) TB has presented new challenges to TB control [1]. Although culture based methods remain the gold standard for diagnosing MDR and XDR TB, it is time consuming and labor intensive due to the slow growth and bio-hazardous nature of *Mycobacterium tuberculosis* [2,3]. Rapid and definitive diagnosis of MDR and XDR TB is desperately needed to quickly initiate proper treatment and effectively control the disease burden. Molecular epidemiological surveillance, functional genomics studies, and more recently, whole genome sequencing in clinical strains have identified genes and mutations in *M*. *tuberculosis* that give rise to resistance to the widely used first and second line antibiotics in the majority of clinical strains [4–7]. Molecular genotypic assays may thus be used as an alternate sensitive and more rapid method to diagnose MDR and XDR TB. These assays can directly detect mutations known to be associated with drug resistance in 80–95% of clinical *M*. *tuberculosis* strains targeting only few genes such as *rpoB*, *katG*, *inhA* promoter, *embB*, *gyrA*, *gyrB*, *rrs*, *eis* promoter, *rpsl* and *pncA* [8–10].

Several molecular assays that detect drug resistance inducing mutations in *M*. *tuberculosis* have been described [11–19]. Recently, considerable attention has been put towards assays utilizing melting temperature (Tm) profiles to identify mutations, owing to their high-throughput nature, ability to be performed in enclosed systems, robustness and rapidity [20–23]. We had previously described a new class of mismatch tolerant Sloppy Molecular Beacon (SMB) probes that enable rapid and high-throughput identification of drug resistance inducing mutations in the *rpoB* RRDR, *gyrA* QRDR, *rrs* gene and the *eis* promoter region of *M*. *tuberculosis* using highly robust and accurate “probe-target hybrid” Tm values [24–26]. A similar dual-labeled probe (DLP) approach has been recently described for first and second-line drug resistance testing. DLP probes are linear probes that also detect mutations by generating Tm profiles of wild type and mutant sequences upon hybridization to amplified targets [21,22]. However, the relative performance of these two assay formats has not yet been evaluated. SMB probes consist of hairpin probe structures that are expected to be thermodynamically more stable than linear probes such as DLPs [27]. In this study, we compared the performance of the SMB and DLP assays with respect to their capability to correctly identify relevant mutations and heteroresistance (a mixture of both wild type and mutant DNA); and also characterized their sensitivity and specificity using pure genomic DNA as well as spiked sputum samples.

## Materials and Methods

### Clinical DNA and mutations analyzed

The assay was performed on 56 clinical *M*. *tuberculosis* DNA samples collected after obtaining written consent from the participants in a study (NCT00341601) conducted at the National Masan Hospital (NMH) in Masan, South Korea and approved by the NMH and National Institute of Allergy and Infectious Diseases institutional review boards. *M*. *tuberculosis* DNA was isolated from the clinical isolates cultured from individual sputum samples by lysis in the presence of InstaGene Matrix (Bio-Rad, USA) and 0.01% Triton X-100 (Sigma) and heating at 90°C for 20 minutes. Supernatant was harvested and 2μL DNA was added to a 20μL PCR reaction for each assay. The DNA samples were chosen to represent clinically significant mutations in the target genes as confirmed by sequencing. The mutations chosen were L511P, Q513K, Q513L, D516L, D516V, D516Y, H526R, H526L, H526D, H526Y, S531W, S531L, L533Q and L533P for the *rpoB* RRDR, G88A, D89N, A90V, S91P, D94N, D94A, D94Y, D94G (GGG and GGC), and D94H for the *gyrA* QRDR, A1401G for *rrs* and G(-10)A, C(-14)T, and G(-37)T for the *eis* promoter. An additional 25 DNA samples with wild type sequence at all the target genes, representing both the Serine (AGC) and Threonine (ACC) polymorphs at the codon 95 of the *gyrA* gene were also chosen as wild type controls.

### Isolation of *M*. *bovis* BCG DNA from spiked sputum samples

Left over sputum samples from non-tuberculosis patients that would have been otherwise discarded were collected from the Microbiology Department at the University Hospital, Newark, NJ, USA. Individual sputum samples were pooled in 30 ml aliquots and each aliquot was confirmed for the absence of *M*. *tuberculosis* using the GeneXpert MTB/RIF assay [28]. Sputa were spiked with serial dilutions of *M*. *bovis* BCG cells to generate final concentrations ranging from 10^6^ CFU/ml to 10^2^ CFU/ml to represent smear positive and smear negative sputum samples. Spiked samples were treated with twice the volume of an in-house sample processing buffer containing NaOH and mucolytic reagents, mixed, incubated briefly, centrifuged and the pellet washed with Phosphate-Buffered Saline solution. BCG DNA was then extracted from the pellet by lysis in the presence of InstaGene Matrix (Bio-Rad, USA) and 0.01% Triton X-100 (Sigma) and heating at 90°C for 15 minutes. Supernatant was harvested and 5μL DNA was added to a 20μL PCR reaction for each assay.

### DNA samples from nontuberculous mycobacteria (NTM) and analytical specificity of the assays

Nineteen NTM isolates from the ATCC repository (Manassas, VA, USA), which included *M*. *abscessus*, *M*. *scrofulaceum*, *M*. *celatum*, *M*. *haemophilum*, *M*. *asiaticum*, *M*. *kansasii*, *M*. *avium*, *M*. *flavescens*, *M*. *szulgai*, *M*. *terrae*, *M*. *fortuitum*, *M*. *intracellulare*, *M*. *marinum*, *M*. *xenopi*, *M*. *thermoresistibile*, *M*. *simiae*, *M*. *triviale*, *M*. *malmoense* and *M*. *smegmatis*, were obtained and used to test the analytical specificity of the assays. DNA was isolated by boiling a loopful of pure culture in InstaGene Matrix (Bio-Rad) with 0.1% Triton X-100 and quantified with a Nanodrop microvolume spectrophotometer (Thermo Scientific). The analytical specificity was tested using 10ng DNA for each PCR.

### DNA sequencing

All DNA samples were subjected to bidirectional Sanger sequencing to confirm the presence or absence of mutations within the target regions. The RRDR of the *rpoB* gene, QRDR of *gyrA*, nucleotides 1293–1537 of the *rrs* gene and a portion of the *eis* coding region including the entire promoter region were amplified using specific forward and reverse primers as described previously [24–26].

### Primers, probes and fluorophore-quencher pair selection

SMB assays were performed with primers as published [24–26] except for the *rpoB* gene, for which we used a new forward primer, rpoB-F2 (5’-ACATCCGGCCGGTGGTCGCC-3’), to enhance the analytical specificity of the assay. The DLP assays were performed with primers and probes as described previously [21,22]. All the DLP and SMB probes were synthesized with sequences as described previously. However, the 5’ fluorophores and 3’ quenchers were changed so that they were maximally compatible with the LightCycler 480 II (Roche Diagnostics Co. Indianapolis, Indiana, USA) emission/excitation filter system and generated the maximum signal intensity from the post PCR melt curves. We replaced the BHQ1 quencher in the rrsP DLP probe with BHQ2 and the ROX fluorophore in the gyraP DLP probe was replaced with Texas Red (TxR). We also similarly changed the fluorophore quencher pairs in our SMB probes. The *rpoB* SMB probes rpo1-TAMRA-BHQ2 and rpo3-FAM-BHQ1 were changed to rpo1-FAM-BHQ1 and rpo3-TxR-BHQ2, *gyrA* SMB probes QDR1-FAM-DABCYL and QDR2-Cy5-BHQ1 were replaced by QDR1-TxR-BHQ2 and QDR2-Cy5-BHQ2 respectively. Primers were obtained from Sigma-Aldrich (St. Louis, Missouri, USA) and probes were obtained from Biosearch Inc (Petaluma, California, USA). The primers and probes used in this assay are listed in S1 Table.

### SMB assays

The SMB assays targeting the *rpoB*, *gyrA*, *rrs* genes and the *eis* promoter region were performed as previously described [24–26]. Each assay plate included 2-4ng of *M*. *tuberculosis* strain H37Rv genomic DNA as positive control, and nuclease-free water (Sigma-Aldrich, St. Louis, Missouri, USA) as assay negative controls. PCR was performed for 50 cycles followed by a melt protocol wherein the samples were first heated at 95°C for 2 minutes followed by cooling down to 45°C and gradual heating to 85°C while monitoring the fluorescence at 1 acquisition per degree centigrade. At the end of the assay, the melt curve profile was analyzed and Tm values were identified using the Light Cycler Tm calling software (Roche Diagnostics Co. Indianapolis, Indiana, USA). One technician manually confirmed each Tm call value.

### Optimization and modification of the PCR conditions for DLP assays

The DLP assays were performed following the protocols as described previously [21,22] with minor modifications as described below. The DLP assay carried out in the studies performed by Luo *et al*. and Liu *et al*. used the a-Taq polymerase enzyme, which lacks 5’ to 3’ exonuclease activity [21,22]. However, this enzyme was not commercially available in US markets at the time of this study. We chose a similar enzyme, Platinum *Tfi* Exo(-) DNA Polymerase (Life Technologies, Norwalk, Connecticut, USA), which also lacks 5’ to 3’ exonuclease activity. All PCR assays were performed on a LightCycler 480 II instrument in 20μL reaction volumes. For the DLP-*rpoB* assay, reaction volume contained 1X *Tfi* PCR buffer, 1.5mM MgCl_2_, 250mM deoxynucleotide triphosphates (dNTPs), 1M Betaine, 400nM target primer, 50nM antisense primer, 200nM each probe, 0.06U/μL of enzyme and 2-5ng of DNA. For the DLP-triplex *rrs*, *eis* promoter and *gyrA* assay, reaction volume contained 1X *Tfi* PCR buffer, 1.5mM MgCl_2_, 250mM dNTPs, 1M Betaine, 1μM of each target primer and 50nM of each antisense primer, 200nM each probe, 0.06U/μL of Platinum *Tfi* Exo(-) Polymerase and 2-5ng of DNA. PCR was performed for 50 cycles followed by a melt protocol wherein the samples were first heated at 95°C for 2 minutes followed by cooling down to 40°C and gradual heating to 85°C while monitoring the fluorescence at 1 acquisition per degree centigrade. At the end of the assay, the melt curve profile was analyzed and Tm values were identified using the Light Cycler Tm calling software (Roche Diagnostics Co. Indianapolis, Indiana, USA). Additionally, one technician manually confirmed each Tm call value generated by the software.

### Assay Limit of Detection (LOD) for the wild type and mutant sequences

Analytical LOD of the DLP and SMB assays for the wild type sequences as well as mutation detection were tested on serial dilutions of wild type DNA samples and different mutant DNA samples corresponding to the genes targeted. Each DNA sample was serially diluted from 2 x 10^5^ genome equivalents to 2 genome equivalents in a ten-fold dilution series and the assays were performed on them ten (for wild type) or five (for mutants) different times. The assay LOD was defined as the minimum DNA input at which the assay unequivocally detected the presence of the mutant or the wild type sequences 100% of the times the assay was performed.

### Detection of heteroresistance

The capacity of the SMB and DLP assays to detect heteroresistance was evaluated by mixing varying ratios of select mutant DNA samples with wild type DNA from Pan susceptible strains and estimating the capacity of the assay to detect the presence of the minimum percentage of the mutant DNA against a wild type DNA background.

### Statistical analysis

Paired and unpaired T-tests were carried out to determine the statistical significance of the comparative performance of the two assays using the online GraphPad software at http://www.graphpad.com/quickcalcs/ttest1/?Format=50 and http://www.graphpad.com/quickcalcs/ttest1/?Format=SD. P-values were calculated to determine the significance of the results obtained.

## Results

### Comparative precision of probe Tm values associated with wild type sequence

We evaluated the reproducibility of the wild type Tm values for each probe type. The rpo1, rpo2, and rpo3 SMB probes generated wild type Tms of 70.2, 70.2 and 66.6°C with standard deviation (SD) values of ±0.15°C, ±0.08°C and ±0.07°C respectively. The DLP rpobP1 and rpobP2 probes generated wild type Tms of 67.7 and 71.5°C, with similar SD values of ±0.08°C and ±0.11°C respectively (Table 1). The QRDR SMB probes generated wild type Tms of 64.3 and 64.4°C with SD of ±0.10°C and ±0.07°C respectively, as compared to the gyraP DLP wild type Tm of 65.8°C with a SD of ±0.24°C, which was significantly larger than the *gyrA* SMB SD values (P <0.0001) (Table 2). The *rrs* SMB probe generated a wild type Tm of 70.3°C with a SD of ±0.15°C compared to the DLP rrsP probe, which resulted in a wild type Tm of 66.5°C and a SD value of ±0.27°C (Table 2), which was significantly larger than the *rrs* SMB SD (P<0.0001). However, the *eis* DLP probe showed a Tm of 57.7°C with SD value of ±0.12°C as compared to the SMB eis1 and eis2 probe wild type Tms of 63.7 and 68.7°C, with SD values of ±0.21°C and ±0.27°C respectively (Table 2), which were significantly larger than the *eis* DLP probe SD (P<0.0001).

**Table 1:** Average dTm values for mutations tested by both DLP and SMB *rpoB* assays.

Mutation	SMB (dTm^b^)(°C)			DLP (dTm^b^) (°C)	
*rpoB* RRDR	rpo1	rpo2	rpo3	rpobP1	rpobP2
L511P	**4.5**	-0.1	0.0	**3.7**	0.0
Q513K	**3.9**	-0.2	-0.1	**4.2**	-0.1
Q513L	**3.4**	0.0	0.0	**3.7**	-0.1
D516L	0.9	**5.3**	-0.2	**9.1**	-0.1
D516V	-0.1	**3.4**	-0.3	**4.4**	0.0
D516Y	1.0	**4.3**	-0.3	**6.0**	-0.2
H526R	0.0	-0.1	**4.8**	0.1	**3.9**
H526L	-0.1	-0.1	**4.9**	-0.2	**3.8**
H526D	-0.1	-0.2	**5.5**	-0.4	**3.7**
H526Y	-0.2	-0.2	**4.2**	-0.2	**3.7**
S531W	-0.2	-0.3	**-2.3**	0.4	**3.6**
S531L	-0.2	-0.5	**-3.7**	-0.2	**3.2**
L533P	-0.3	-0.2	**-1.3**	0.1	**3.5**
WT Tm StDev^a^	±0.15	±0.08	±0.07	±0.08	±0.11

^a^ Standard Deviations were calculated from an average of 20–40 separate reactions for each probe

^b^ *dT*_*m*_ = WT *T*_*m*_—mutant *T*_*m*_

The dTm values identifying the mutations are shown in bold

**Table 2:** Average dTm values for mutations tested by both DLP and SMB *gyrA*, *eis* promoter and *rrs* assays.

	SMB (dTm^b^)(°C)		DLP (dTm^b^) (°C)
**gyrA QRDR**	**QDR1**	**QDR2**	**gyraP**
G88A	**2.3**	**6.0**	0.7
D89N	**5.1**	**6.0**	**7.7**
A90V	**-3.9**	**-4.0**	**6.2**
S91P	**4.9**	**4.4**	**7.5**
D94G (GGC)	**-4.4**	**1.9**	**2.4**
D94N	**1.1**	**5.8**	**7.5**
D94A	0.0	**4.4**	**3.2**
D94Y	**1.3**	**5.8**	**7.3**
D94G (GGG)	**-2.2**	**4.4**	**3.8**
D94H	**1.2**	**6.1**	**7.0**
95(Thr)	**0.0**	**0.0**	**0.0**
95(Ser)	**2.4**	**1.2**	0.2
WT Tm StDev^a^	±0.10	±0.07	±0.24
***eis* promoter**	**eis1**	**eis2**	**eispP**
G(-10)A	0.3	**4.2**	**6.4**
C(-14)T	0.1	**6.3**	**4.9**
G(-37)T	**4.7**	0.1	**5.0**
WT Tm StDev^a^	±0.21	±0.27	±0.12
**rrs**	**rrs-SMB**		**rrsP**
A1401G	**3.9**		**0.9** ^#^
WT Tm StDev^a^	±0.15		±0.27

^a^ Standard Deviations were calculated from an average of 20–40 separate reactions for each probe

^#^ result represents an average of 22 separate reactions.

^b^ *dT*_*m*_ = WT *T*_*m*_—mutant *T*_*m*_

The dTm values identifying the mutations are shown in bold

### Comparative performance of mutation detection

The performance of the DLP and the SMB *rpoB* assays was compared using a panel of 29 samples representing 13 different mutations in the *rpoB* RRDR (Table 1). For the SMB assay, a Tm difference (dTm) value was considered significant for mutation detection only if it was ≥15 times the SD value of the wild type Tm for that individual probe. For the mutations spanning the codons 511–516, the rpo1 and rpo2 SMB probes generated distinct Tm shifts (dTm) ranging from 3.4°C to 5.3°C. For the mutations spanning the codons 526 to 533, the rpo3 SMB generated three different ranges of codon specific dTms, namely 4.2 to 5.5°C for codon 526 mutations, -2.3°C to -3.7°C for codon 531 mutations and -1.3°C for the L533P mutation (Table 1). Thus the SMB assay specifically identified the mutations at codons 526, 531 and 533. The DLP chemistry on the other hand uses a single fluorophore to label both the *rpoB* probes. We found that the DLP assay generated wild type Tms of 67.7 and 71.5°C from the two probes which resulted in a merged double peak, consistent with the previous study [22]. This merged peak diverged into two distinct double peaks for the mutations spanning codons 511 to 516 due to a Tm shift of 3.7 to 9.1°C from the probe rpobP1, which was consistent with the previous findings and enabled clear identification of the mutations in the codons 511–516 [22]. This was comparable to the performance by the SMB probes. However, for all the mutations spanning the codons 526–533, the DLP assay resulted in a Tm shift of 3.2 to 3.9°C from rpobP2 and no Tm shift from rpobP1 causing the melt peaks of the two probes to overlap into a single peak with a Tm of 67.8°C, which was identical to the rpobP1 wild type Tm of 67.7°C (Table 1 and S1 Fig). This peak pattern did identify the mutations at the codons 526, 531 and 533, but it could not distinguish between them unlike the SMB assay.

A representative panel of 17 samples was chosen with 10 individual *gyrA* QRDR mutations (Table 2) to compare the performance of the SMB and the DLP *gyrA* assays. All the mutant DNA samples were Threonine polymorphs at the codon 95 (ACC). The two-probe SMB assay successfully identified all 10 mutants, generating a dTm that was at least 15-times the SD of the WT Tm values for at least one of the two *gyrA* QRDR SMB probes. The dTm values for at least one of the two probes ranged from -2.2 to -4.4°C and from +2.4 to +6.1°C depending on the position of the mutation within the QRDR (Table 2). The wide range of dTms generated by the two *gyrA* SMB probes enabled selective identification of G88A, D89N, A90V, S91P, D94G (GGC), D94A, and D94G (GGG) mutations (Table 2), which might be useful for distinguishing between high and low levels of resistance to fluoroquinolones [29–31]. While performing the *gyrA* DLP assay, we observed that the concentrations of the excess and the limiting primers as described in the paper by Liu *et al*. [21] generated an excess of the non-target strand. The resulting assay did not produce any melt curves. Once the primer ratio was reversed, the assay performed as expected. We concluded that the published primer ratios were mistakenly reversed, and performed all subsequent experiments with the corrected primer ratios. The gyraP DLP assay also successfully identified 9/10 mutations tested, with dTms ranging from 2.4 to 7.7°C. However, the assay failed to identify the G88A mutation with a high degree of accuracy, as it generated a dTm of only 0.7°C (Table 2). This is only 2.9X times the standard deviation of the probe, which may not be suitable in crude DNA preparations from clinical samples which could generate higher SD values for the wild type Tms (Table 2). This is likely due to the fact that the gyraP probe does not bind to the first two nucleotides of codon 88 and thus does not directly hybridize to the mutant SNP. The DLP assay also generated identical dTm values for mutations at the codons 89, 90 and 91, as well as several mutations at codon 94 (Table 2) which prevented specific identification of these mutations, although D94G (GGC) could be specifically identified (Table 2). Wild-type samples containing both the *gyrA* codon 95 Serine (AGC) and Threonine (ACC) polymorphs were chosen to evaluate the ability of both assays to detect these neutral mutations. The *gyrA* codon 95-Threonine polymorph was used as the wild-type reference sequence as our mutation panel contained only Threonine-95 polymorphs. The SMB assay detected each polymorph specifically as individual wild type polymorphs and clearly differentiated them from all the different mutations tested, while the DLP assay detected both as wild-type with identical Tms (Table 2).

Three *eis* promoter mutations G(-10)A, C(-14)T, and G(-37)T, were tested using both the SMB and DLP assays. All mutations were successfully detected by both the SMB and DLP *eis* promoter assays. The 2-probe SMB assay generated dTms ranging from 4.2 to 6.3°C, with the eis1 SMB probe identifying G(-37)T, and the eis2 SMB probe identifying G(-10)A and C(-14)T mutations. The DLP eispP probe also showed dTms ranging from 4.9 to 6.4°C, identifying the same mutations with a performance comparable to the SMB probes (Table 2).

A total of 22 clinical DNA samples with the *rrs* A1401G mutation were tested with both the SMB and DLP *rrs* assays. The SMB assay clearly identified all the A1401G mutant samples tested, but the DLP approach failed to detect 10/22 samples as mutants, due to the lack of any definitive dTm. The average Tm values of the *rrs* SMB probe for the wild type and mutant sequences were 70.3°C and 66.4°C respectively producing a robust average dTm value of 3.9°C (Table 2). On the other hand, the average Tm values of the rrsP DLP were 66.4°C and 66.1°C for the wild type and the mutant sequences respectively, with a relatively low average dTm value of 0.9°C (Table 2). Additionally, the mutant Tm values for the rrsP DLP probe were highly variable showing a Tm SD value ±0.93°C.

### Analytical sensitivity of each assay using genomic DNA

**Fig 1:**
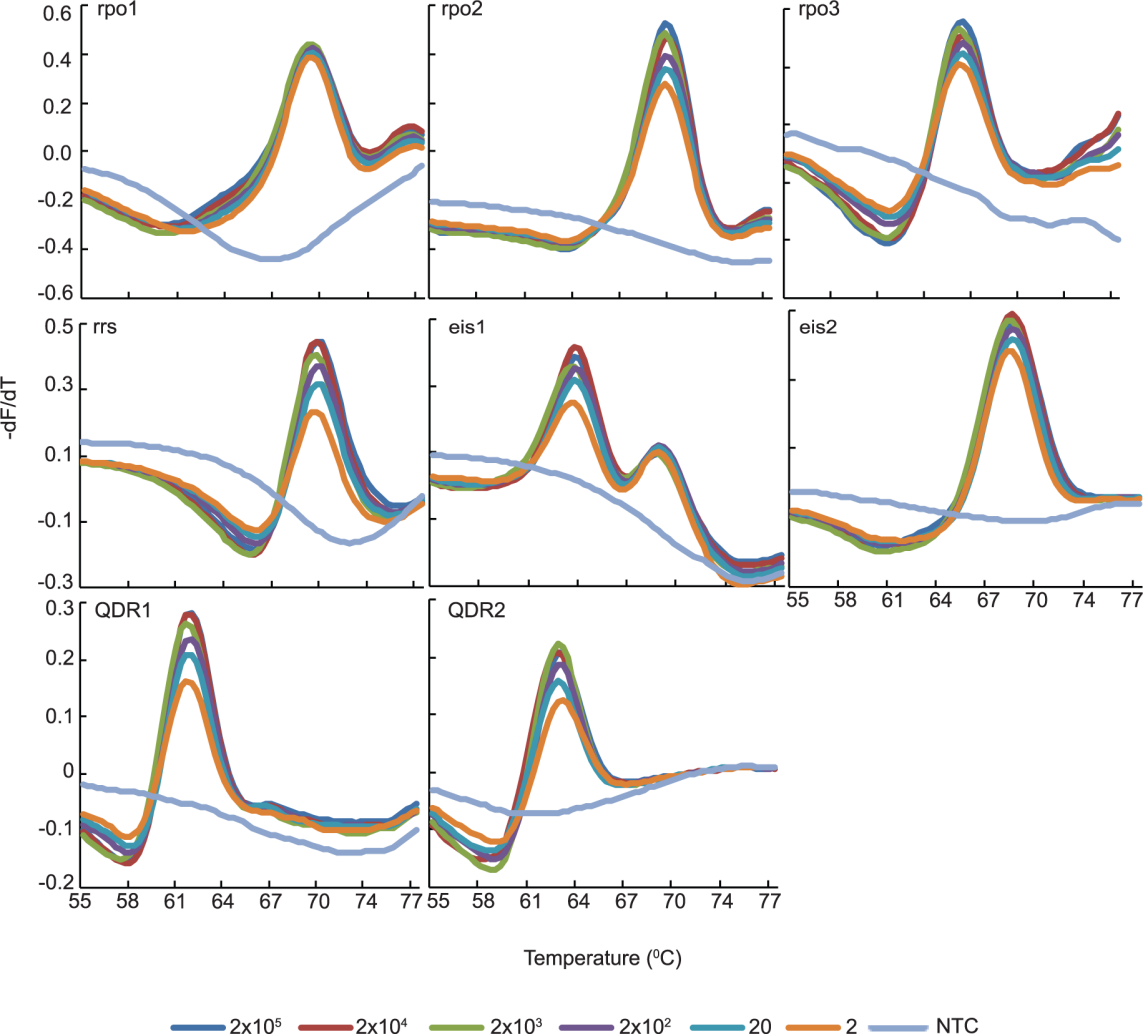
Analytical sensitivity of the SMB assays. Melting temperature first derivative peak profiles of the targets tested for the SMB probes on serial dilutions of *M*. *tuberculosis* H37Rv genomic DNA. Input DNA amount is indicated as genome equivalents.

**Fig 2:**
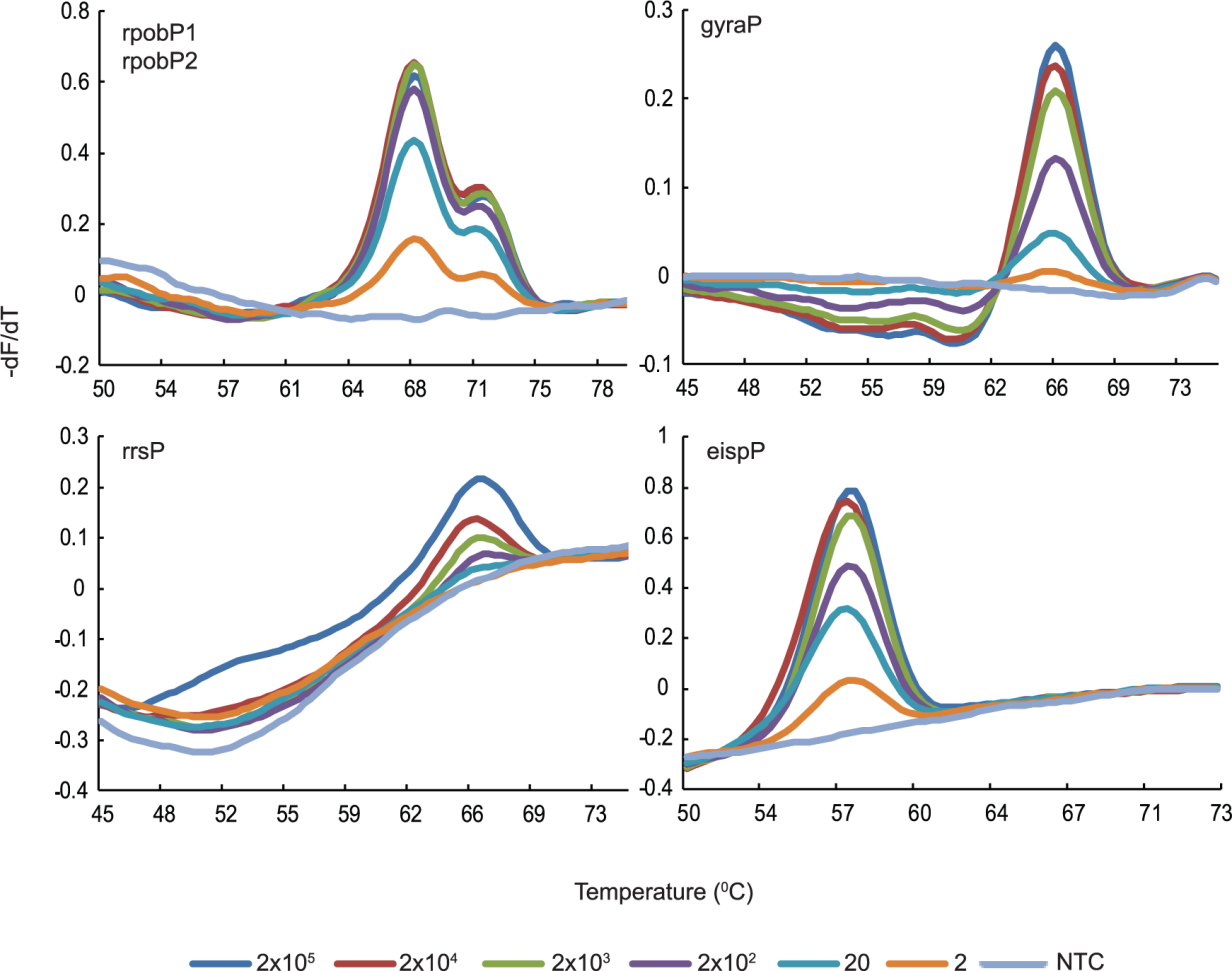
Analytical sensitivity of the DLP assays. Melting temperature first derivative peak profiles for all the targets for the DLP probes on serial dilutions of *M*. *tuberculosis* H37Rv genomic DNA. Input DNA amount is indicated as genome equivalents.

**Fig 3:**
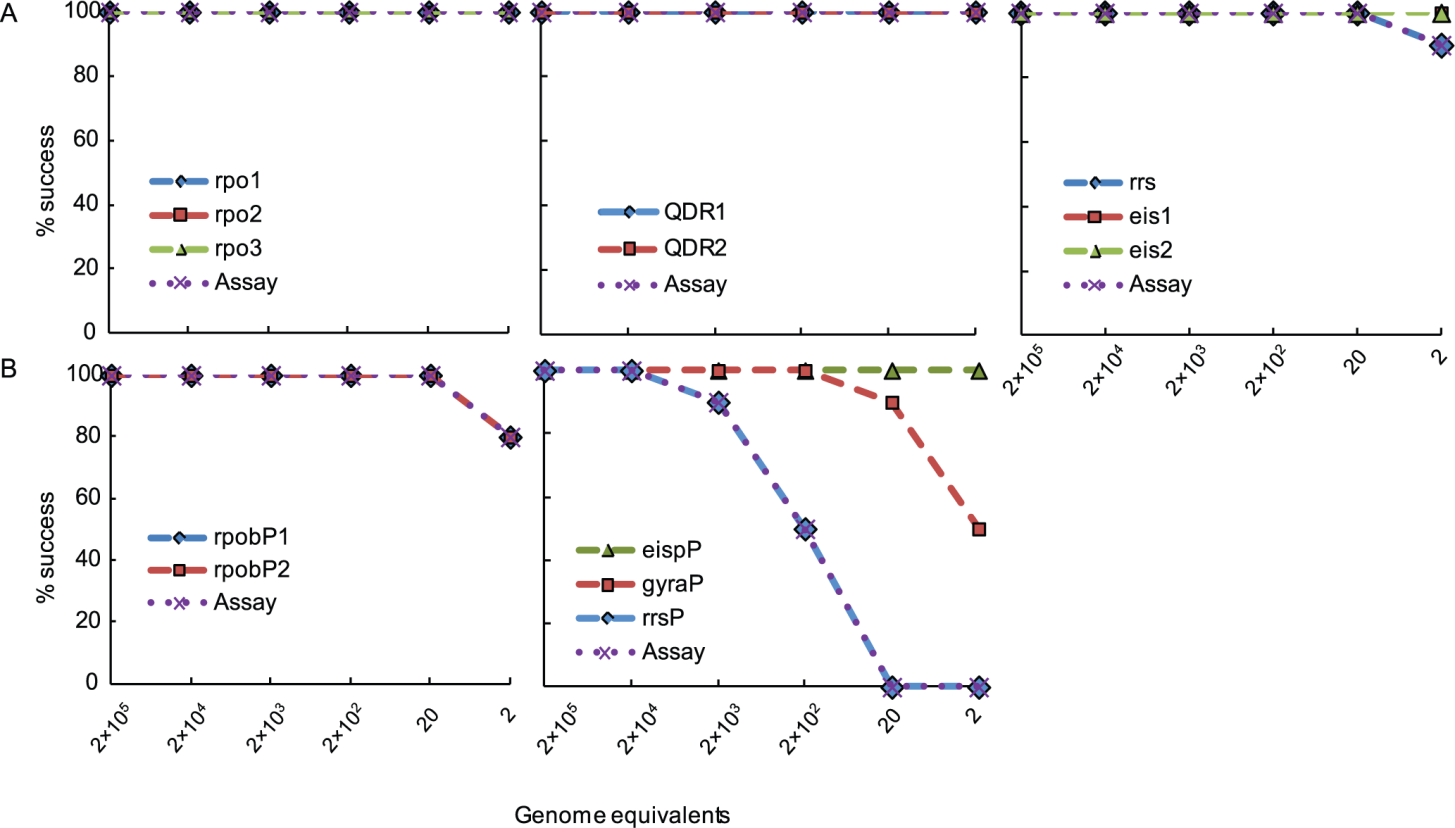
Limit of detection (LOD) of the individual SMB and DLP assays on *M*. *tuberculosis* H37Rv genomic DNA. Assays were performed with a dilution series of genomic equivalents as indicated. Assay success rates for individual probes and the entire assay over the input DNA dynamic range are shown. Panel (A) SMB probe and assay performance; Panel (B) DLP probe and assay performance.

We analyzed the analytical sensitivities of both assays for all the four drug resistance target genes under study using serial dilutions of genomic DNA (gDNA) from *M*. *tuberculosis* strain H37Rv ranging from 2 x 10^5^ to 2 genomic equivalents. Each assay was run in ten experimental replicates for both SMB and DLP assay formats over the entire concentration of DNA used. Over the entire dynamic range, the SMB assay (Fig 1) generated more robust peaks than the DLP assay (Fig 2), especially for the low quantity input DNA template. The LOD was defined as the input DNA for which the assay generated the correct Tm values from all the probes 100% of the times performed. The SMB assay LOD was 2 genome equivalents for each of the *rpoB*, *gyrA* and the *eis* promoter assays, and 20 genome equivalents for the *rrs* assay (Fig 3A). The DLP assays on the other hand resulted in LODs of 20, 200, 2 x 10^4^, and 2 genome equivalents for the *rpoB*, *gyrA*, *rrs* and the *eis* promoter assays respectively (Fig 3B). Thus, except for the *eis* promoter assay where the LOD of the SMB and the DLP assays were comparable, the SMB assay showed a superior analytical sensitivity over the DLP assay.

### Assay sensitivity with DNA isolated from sputum spiked with BCG

**Fig 4:**
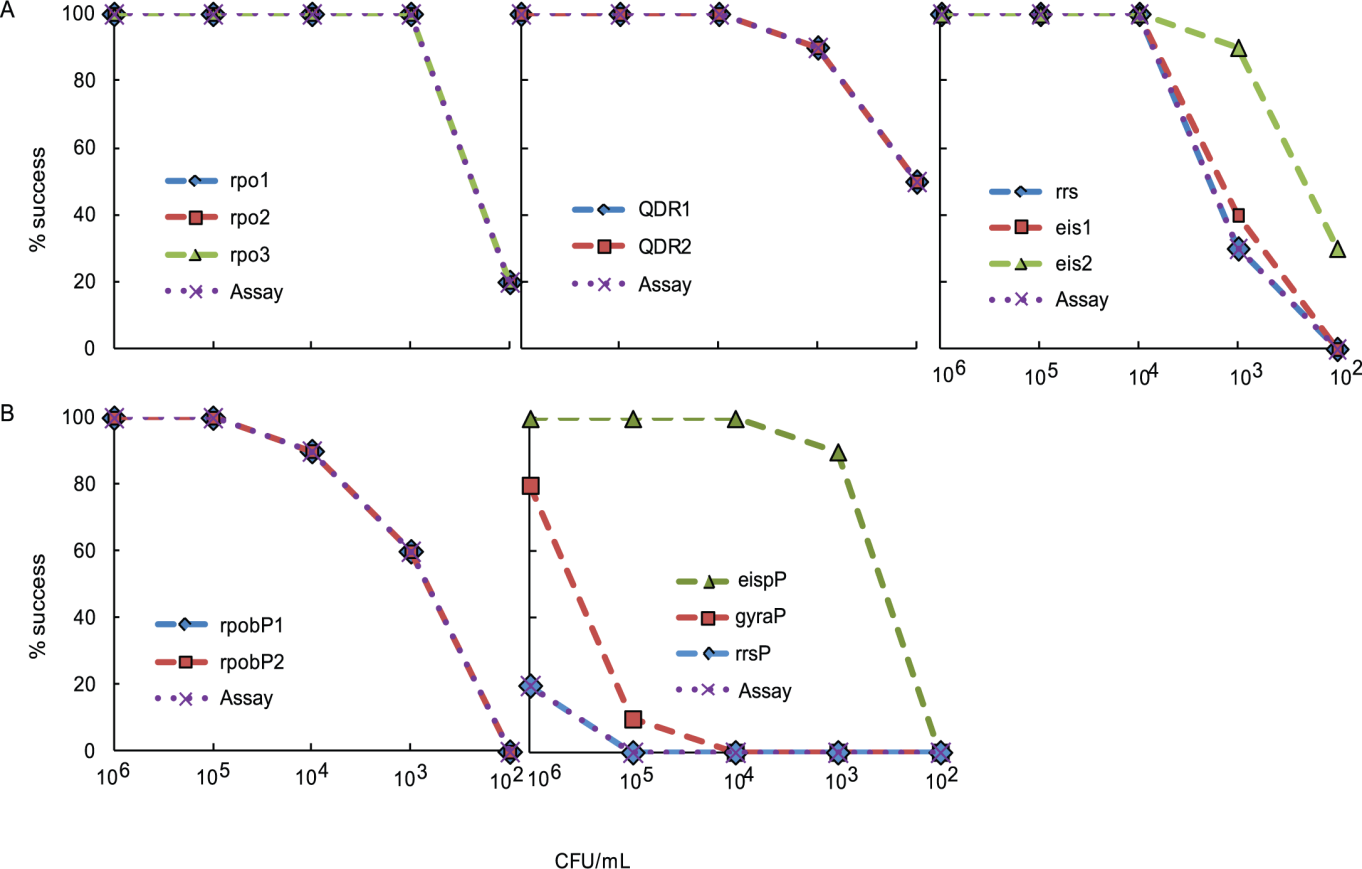
Limit of detection (LOD) of the individual SMB and DLP assays on sputum spiked with *M*. *bovis* BCG CFU. Both SMB and DLP assays were performed on DNA isolated from sputum spiked with a serial dilution of *M*. *bovis* BCG CFU as indicated. Assay success rates for individual probes and the entire assay over the dynamic range of spiked CFU/ml are shown. Panel (A) SMB probe and assay performance; Panel (B) DLP probe and assay performance.

We also compared the sensitivities of the DLP and SMB assays testing DNA extracted from *M*. *bovis* BCG CFU spiked in *M*. *tuberculosis* negative sputum to simulate clinical samples, with concentrations ranging from 10^6^ to 10^2^ CFU/mL for a total of five experimental replicates. The SMB assay LOD for the *rpoB* assay was 1,000 cfu/ml and that of the *gyrA*, *rrs*, and *eis* promoter assays were 10^4^ CFU/mL while the LOD of the *rpoB* DLP assay was 10^5^ CFU/ml, >10^6^ CFU/ml for the both the *gyrA* and the *rrs* assays, and 10^4^ CFU/mL for the *eis* promoter assay (Fig 4, panels A and B). The SMB assay was thus 2 logs more sensitive for *rpoB* assay, at least 2 logs more sensitive for *gyrA* and *rrs* assay, and was comparable to the DLP *eis* promoter assay in these tests of spiked sputum samples. These results are consistent with the LODs that we had determined using pure *M*. *tuberculosis* genomic DNA.

### Sensitivity for mutation detection

#### *rpoB* RRDR

**Fig 5:**
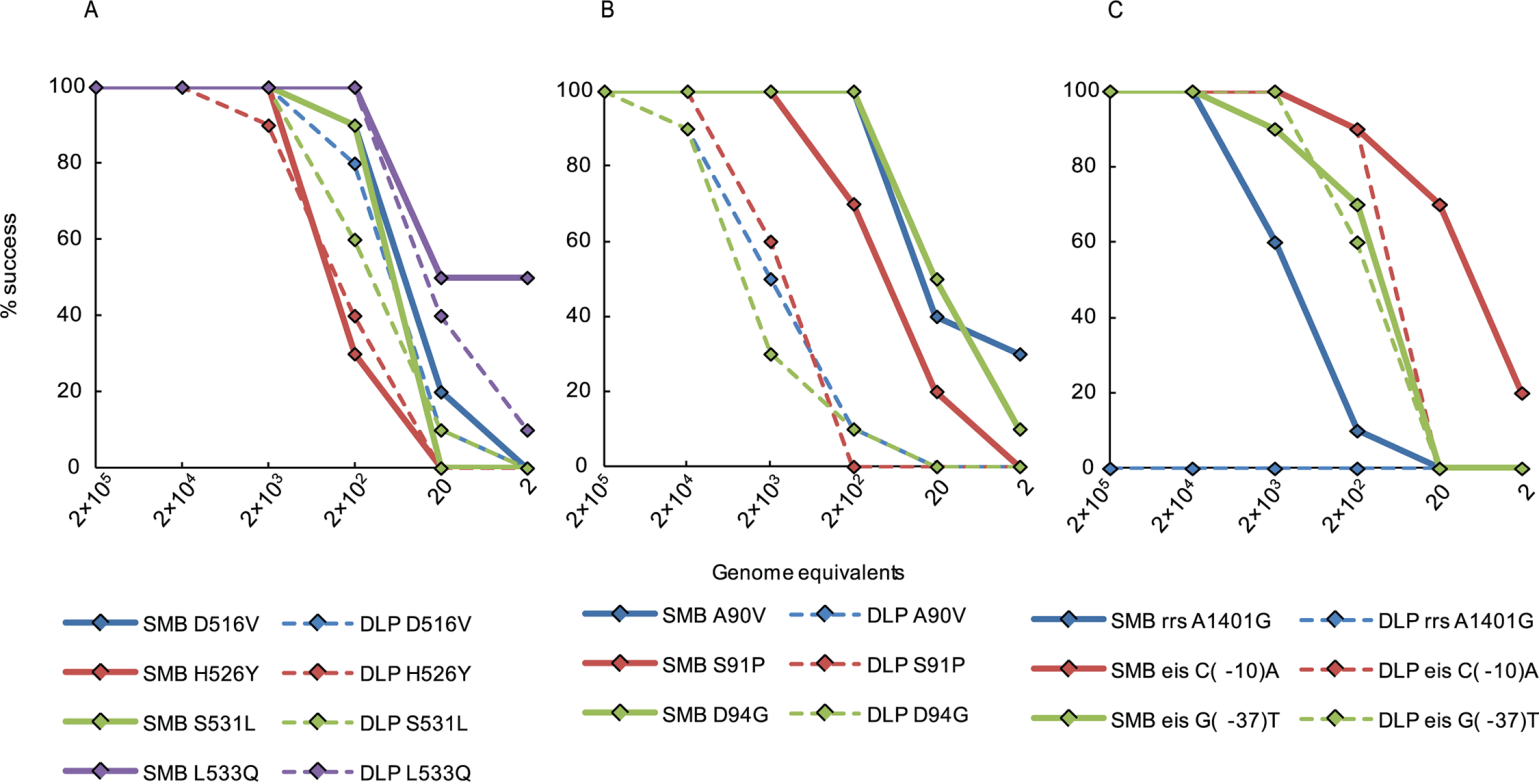
Analytical sensitivity of SMB and DLP probes on mutant DNA from clinical samples. Success rates for the detection of selected mutants by the SMB and the DLP assays are shown. Panel (A) *rpoB* mutants; panel (B) *gyrA* mutants and panel (C) *rrs*/*eis* mutants. Solid lines show SMB assay performance and dotted lines show DLP assay performance.

We chose four of the most common *rpoB* RRDR mutations; D516V, H526Y, S531L, and L533Q to test the analytical LOD for mutation detection. Both the DLP and the SMB assays detected 2 × 10^3^ genomes of D516V mutant DNA 100% of the time (Fig 5). The SMB assay showed a log better LOD for the H526Y mutation, detecting it down to 2 × 10^3^ genomes vs. 2 × 10^4^ genomes for the DLP assay. Both the SMB and DLP *rpoB* assays detected the S531L mutation in 100% of replicates at 2 × 10^3^ genomes, but the SMB assay detected the this mutation 90% of the time at 200 genomes DNA input compared to the DLP assay, which detected this mutation 60% of the time at 200 genomes (P = 0.0007) (Fig 5, Panel A). For the L533Q mutation, both the DLP and the SMB assays showed an identical LOD of 200 genome equivalents, but the SMB assay had higher percent success at 2 genomes input than the DLP assay (50% vs. 10%; P<0.0001) and generated more robust melt peaks at the LOD (S2 Fig). Overall the SMB *rpoB* assay showed a log better LOD for the *rpoB* H526Y mutation compared to the DLP approach and although the 516, 531, and 533 mutations had comparable LODs the SMB assay was more frequently positive at the sub-LOD range (Fig 5, Panel A).

#### *gyrA* QRDR

Three common mutations in the QRDR were chosen to assess the LOD of mutation detection for the SMB and DLP *gyrA* assays as follows: A90V, S91P, and D94G (GGC). For all the mutations tested, the SMB assay showed greater sensitivity than the DLP assay. The LOD for the A90V and D94G (GGC) mutations for the SMB assay was three logs better than the DLP assay, detecting the mutations 100% of the time with a DNA input of 200 genomes vs. 2 × 10^5^ genomes for the DLP assay (Fig 5, Panel B). The SMB assay also showed a log better LOD for the S91P mutation (2 × 10^3^ genomes) as compared to the DLP assay (2 × 10^4^ genomes) (Fig 5, Panel B).

#### *eis* promoter assay

Mutation detection sensitivity was tested for both SMB and DLP assays by analyzing *eis*(-10) and *eis*(-37) promoter mutations. The SMB and DLP *eis* assay LODs were comparable for the *eis*(-10) mutation, as both assays detected the mutation at 2 × 10^3^ genomes input mutant DNA 100% of the time (Fig 5, Panel C). However, at lower input DNA concentrations, the SMB assay detected the *eis* (-10) mutation more reliably than the DLP assay (Fig 5, Panel C). When we tested the *eis* (-37) mutation, the DLP assay showed an LOD of 2 × 10^3^ genomes which was a log better than the SMB assay LOD of 2 × 10^4^ genomes (Fig 5, Panel C).

#### *rrs* assay

The SMB *rrs* assay showed significantly better sensitivity than the DLP *rrs* assay for detecting the A1401G mutation. The SMB assay detected the mutation 100% of the times at 2 × 10^4^ genomes of input DNA and 60% and 10% of the time at 2 × 10^3^ and 200 genomes of input DNA respectively (Fig 5, Panel C). The DLP *rrs* assay however, failed to detect the presence of the A1401G mutation even when 2 × 10^5^ genomes of the mutant DNA were added to the assay (Fig 5, Panel C).

### Sensitivity of heteroresistance detection

We compared the heteroresistance detection capability of the two assays by mixing mutant and wild-type DNA populations of selected mutations for all the three gene targets. DNA mixtures tested contained zero to 100% of mutant DNA in 10% increments against a background of wild type DNA to mimic the wide range of heteroresistant mixtures that may be present in clinical samples.

#### *rpoB* RRDR

We selected four mutations within the RRDR; D516L, D516V, H526Y, and S531L to analyze the heteroresistance detection capacity of the two assays. The SMB *rpoB* assay was less sensitive than the DLP assay in detecting mixtures with D516L and D516V mutations, as it detected mixtures with 60% mutant DNA and failed to detect mixtures with 50% mutant DNA and lower, whereas the DLP *rpoB* assay detected these mutations down to 30% mutant DNA present (Table 3). Neither the SMB nor the DLP *rpoB* assay detected the *rpoB* H526Y mutation in mixtures of mutant and wild-type DNA. The SMB *rpoB* however, was more sensitive in detecting the S531L mutation in a mixture of wild type and mutant DNA, clearly detecting the presence of as low as 10% S531L DNA mixed with 90% WT DNA, compared to 40% mutant DNA detected by the DLP *rpoB* assay (Table 3).

**Table 3:** Heteroresistance detection. Comparative detection success at different concentrations of mixed wild-type and representative mutant DNA.

Target gene	Mutation	Minimum percentage of mutant sequence detected	
		SMB	DLP
***rpoB***	**D516L**	60%	**30%**
	**D516V**	60%	**30%**
	**H526Y**	100%	100%
	**S531L**	**10%**	40%
***gyrA***	**A90V**	**10%**	60%
	**S91P**	30%	30%
	**D94N**	**70%**	80%
	**D94G (GGC)**	**10%**	100%
	**D94A**	**30%**	50%
***rrs***	**A1401G**	**70%**	100%
***eis***	**C(-10)A**	40%	**10%**
	**G(-37)T**	40%	**30%**

Minimum percentages of the mutant detected by an individual assay as compared to the other are shown in bold.

#### *gyrA* QRDR

The ability of each assay to detect *gyrA* heteroresistant populations was determined by using 5 different mutant DNA sequences; A90V, S91P, D94N, D94G (GGC), and D94A in mixtures with wild-type sequence as mentioned previously. Except for the S91P mutation, the SMB assay detected all the other *gyrA* mutant DNA mixtures with higher sensitivity than the DLP gyraP probe (Table 3). The SMB assay detected down to 10, 70, 10 and 30% mutant A90V, D94N, D94G and D94A respectively, compared to the DLP assay which required the mutations to be present at 60, 80, 100 and 50%, respectively. S91P DNA was detected by both assays with equal sensitivity at 30% mutant DNA. The SMB QRDR assay was thus more sensitive in detecting fluoroquinolone heteroresistance for 4/5 of the most common QRDR mutation types.

#### *eis* promoter

Mixed samples containing C(-10)A and G(-37)T *eis* promoter mutations were detected more efficiently using the DLP *eis* assay, allowing the detection of 10% mutant DNA for the C(-10)A mutation and 30% mutant DNA for the G(-37)T mutation (Table 3). In contrast, the SMB *eis* assay detected C(-10)A and G(-37)T mutations in mixtures when at least 40% mutant DNA or more was present

#### *rrs* A1401G

The SMB *rrs* assay was more efficient at detecting the A1401G mutation in a mixed DNA population. The DLP *rrs* assay was unable to detect the mutant sequence at all in mixed DNA preparations (Table 3). In contrast, the SMB *rrs* assay could detect 70% A1401G DNA mixed with 30% WT mutant sequence.

### Analytical Specificity of the SMB and DLP assays

The analytical specificity of the two assays was tested on a panel of 19 species of non-tuberculosis mycobacteria (NTM) using 10^6^ genomic equivalents of DNA (S1 Table). The SMB *rpoB* assay generated Tms from only 4/19 different NTM species. In comparison the DLP *rpoB* assay generated Tms in the presence of 13/19 species (S2 Table). None of the Tms generated by the SMB *rpoB* assay from the 4 NTM species corresponded to any wild type or mutant Tm code produced by *M*. *tuberculosis*. The DLP assay also generated Tm values from NTM species which were distinct from the Tms of the WT or mutant *rpoB* sequences of *M*. *tuberculosis*. The SMB and DLP *eis* assays were both highly specific, generating Tms only from wild-type H37Rv gDNA. The DLP *gyrA* assay produced a Tm from *M*. *avium*, while the SMB QRDR2 probe generated a Tm from both *M*. *abscessus* and *M*. *marinum*. However, these Tms were completely distinct from either the wild type or the mutant Tms and the QRDR1 probe did not generate any Tms for these species (S2 Table). The SMB *rrs* assay was designed so that the primers were specific to the Mycobacterium genus as the sequence flanking nucleotides 1401 and 1402 are highly conserved. All the NTMs generated a wild-type Tm except for *M*. *xenopi*, which did not generate any Tm (S2 Table). The DLP *rrs* assay generated a Tm of 67.9°C from *M*. *avium*, and no other NTM species tested. This may be due to the poor analytical sensitivity of the DLP *rrs* assay.

### Effect of fluorophore and quencher selection on probe performance

We analyzed the effect of changing the fluorophore-quencher pair on performance of the DLP probes, since the fluorophore-quencher pairs used in some of the DLP probes in our study were different from those described in the studies by Liu *et al*. [21]. We selected the *rrs* probe for this analysis, since in our study the *rrs* DLP assay performed less well than the SMB assay. Our version of the *rrs* DLP probe contained a BHQ2 quencher instead of BHQ1 as described in the original study [21], since BHQ2 is known to be a better quencher than BHQ1 for Cy5 dyes [32] and thus expected to generate better signal to noise ratio. We also evaluated the performance of the *rrs* DLP probe linked to a BHQ1 quencher as described in the original study [21]. A new *rrs* SMB probe with a FAM-BHQ1 fluorophore quencher pair (instead of Cy5-BHQ2) was also made to test for effects on the probe performance when both fluorophore and quencher were changed. The SMB *rrs* probes performed identically, regardless of fluorophore-quencher pair, with an identical LOD of 2 genomes and an unchanged average dTm of 3.9°C for mutation detection. However, changing the BHQ2 quencher on the *rrs*-DLP probe to a BHQ1 quencher further degraded the performance of the DLP *rrs* assay. The Cy5-BHQ1 *rrs* probe failed to generate any measurable Tm peaks from the wild type and the mutant DNA tested (S3 Fig). These results indicate that our use of a BHQ2 quencher would be preferred for the *rrs* DLP probe, at least in the LC480 Real time PCR system used in our study.

## Discussion

Our approach and that of Liu *et al*. and Luo *et al*. have been shown to be among the promising real-time PCR methods to detect drug resistance in *M*. *tuberculosis* [21,22,24–26]. Given the potential applicability of these assays across a variety of detection platforms, we believed that it would be useful to carefully compare and contrast their relative benefits. With regards to mutation detection, we observed that the two assays had similar overall abilities to detect mutations in the *rpoB*, *gyrA* and the *eis* promoter targets associated with resistance to Rifampin, the Fluoroquinolones, and low level Kanamycin resistance, respectively. However, the SMB assay was better at specifically identifying and differentiating mutations in *rpoB* codons 526, 531 and 533. Each mutation generated a specific Tm “code” that was unique to that codon in the SMB assay. Mutations in *rpoB* 531 codon are associated with high level Rifampin resistance, while 533 mutations are associated with lower level resistance or produce discordant results compared to phenotypic DSTs [33,34], which makes it beneficial to specifically identify these mutations.

The SMB assay uses three different probes to target the *rpoB* gene while the one-color DLP *rpoB* assay uses two different probes linked to the same fluorophore. Although the one color-two probe strategy is potentially more cost effective and has the advantage that it can be multiplexed with other probes, this strategy decreases the capacity to identify specific mutations. Additionally, the DLP probes do not span the entire *rpoB* RRDR and would miss any mutations between RRDR codons 507–510 and 520–525, although they are clinically rare. Although not specifically addressed in our study, we predict that the single merged peak produced by the DLP assay for the *rpoB* mutations in the codons 526, 531 and 533 (S1 Fig) will be indistinguishable from the single rpobP1 peak produced by a wild type *rpoB* sequence in cases where the rpobP2 DLP probe is accidentally omitted from the assay. This risk needs to be considered when devising internal assay controls or quality assurance processes for manufactured assays. Similarly the SMB *gyrA* assay specifically distinguished the *gyrA* mutations at the codons 90, 91 and 94, which the DLP assay was unable to do. Recent studies indicate that specific identification of *gyrA* QRDR mutations may provide clues to high and low level fluoroquinolone resistance [35].

We found that both assays detected mutations in the *eis* promoter quite well. Although the DLP and the SMB assays generated comparable dTm values, detection of heteroresistance at this locus was better in case of the former as shown in this study. Furthermore, the SMB assay used two different probes to detect *eis* promoter mutations whereas the DLP assay accomplished this with a single probe. In contrast, SMB assay was significantly better at detecting mutations in the *rrs* target associated with higher level Amikacin and Kanamycin resistance. We found that the DLP assay failed to distinguish *rrs* wild type and the mutant sequences in as many as 10/22 tests of mutant *rrs* DNA. An *in-silico* analysis of DLP probe hybridizations to wild type and mutant *rrs* sequences suggested that the A to G mutation at *rrs* position 1401 would be predicted to produce a relatively low dTm due to a G-T base pair between the mutant sequence and the probe. We circumvented this problem in the SMB assay by designing the *rrs* SMB probe in the opposite strand which resulted in an A-C mismatch between the probe and the mutant sequence and a resulting large dTm.

Mutation detection in an analytic setting should not be the only standard for judging assays intended for use on clinical samples. Assays must also be able to perform well in the presence of very small numbers of targets. We found that the analytical sensitivity of the SMB assay was greater than that of the DLP assay when tested against both wild type and mutant genomic DNA for almost all drug resistance mutations and targets. Only the *eis* promoter assay had comparable sensitivity. The SMB assays were also more sensitive when the DNA was isolated from sputum samples spiked with BCG. For the *rpoB*, *gyrA*, and *rrs* assays, the sensitivity of the SMB assay was at least two logs more sensitive than the DLP assay. The SMB assay was also much better at detecting heteroresistance at most sites except for *eis* promoter mutations and *rpoB* mutations at codon 516. However, it should be noted that the DLP *gyrA*, *rrs* and *eis* assays were performed in triplex reactions as described by the authors, while SMB *gyrA* and the *rrs-eis* assays were performed in uniplex and duplex formats respectively. Uniplex assays often have better analytical sensitivities than multiplex assays [36], which may explain the differences which we observed.

This study has two limitations; first, we could not exactly reproduce the assay conditions and PCR composition of the DLP assay as described. We found that the fluorophore-quencher pair used for the *rrs* DLP probe by Liu *et al*. [21] performed very poorly when tested using the Roche LC480 platform in our study. Therefore, we created an *rrs* DLP probe with a different fluorophore quencher pair that we believed to be more optimal. This and other fluorophore-quencher changes were made to the DLP assay in order to make the fairest comparison possible between the DLP and SMB assay when tested on the Roche LC480 platform. We also used a different Taq Polymerase for the DLP assay, because the described polymerase was not available to us in our United States laboratory. However, the Taq Polymerase selected had identical processivity to the one described. Second, we found that we needed to reverse the primer ratios of the DLP *gyrA* assay, changing the limiting primer into the excess primer, and vice versa. When we used the published primer ratio, the DLP *gyrA* assay failed completely in our hands. An *in silico* analysis of the DLP *gyrA* assay indicated that the limiting primer (as described) produced single stranded target that was identical rather than complementary to the DLP *gyrA* assay probe. The DLP assay improved dramatically when we corrected what we believed was a simple mistake in the previously published paper; and we used this corrected assay in all of our study comparisons.

In summary, our comparison of these two molecular DST assays for detection of Rifampin, Fluoroquinolones, Amikacin, and both high-level and low-level Kanamycin resistance demonstrates the relative advantage of each assay for certain laboratory defined conditions. The SMB assay performed better overall, especially in terms of assay sensitivity and LOD. However, several advantages of the DLP assay were also noted. The choice of which assay to use will clearly depend on the testing platform and its intended use. Our study also demonstrates what we believe to be an appropriate and accurate way to compare molecular DST assays for *M*. *tuberculosis* in a laboratory setting. Given the cost of performing clinical trials, we suggest that our approach could be widely implemented to study existing and future molecular tests, with only the best assays selected for advancement to large scale clinical investigations.

## Supporting Information

S1 Fig DLP *rpoB* assay melt curves obtained from wild type sequence and select mutations at codons 526, 531 and 533. WT implies “Wild type”.
(EPS)

S2 Fig Melt peak profiles on serial dilutions of *M*. *tuberculosis* DNA carrying the L533Q mutation. Panel A, SMB assay and Panel B, DLP assay. Numbers represent the DNA genome equivalents added to the reaction.
(EPS)

S3 Fig Melting profiles on serial dilutions of H37Rv DNA using the rrsP DLP probe. Panel (A) Cy5-BHQ1 and panel (B) Cy5-BHQ2 fluorophore-quencher combinations. NTC implies “No template control.”
(EPS)

S1 Table Primer and probe sequences and the fluorophore/quencher pairs used in the SMB and DLP assays. (DOCX)

S2 Table Analytical specificity of the SMB and DLP assays for the different gene targets. (DOCX)
