# Supplementary material for: Comparative Evaluation of Sloppy Molecular Beacon and Dual-Labeled Probe Melting Temperature Assays to Identify Mutations in Mycobacterium tuberculosis Resulting in Rifampin, Fluoroquinolone and Aminoglycoside Resistance
Source: PLoS One. 2015 May 4;10(5):e0126257. doi: 10.1371/journal.pone.0126257 (PMC4418795; doi:10.1371/journal.pone.0126257)
Supplement: S1 Table — (DOCX) [file pone.0126257.s004.docx]

**Table S1.**  Primer and probe sequences and the fluorophore/quencher pairs used in the SMB and DLP assays.

| **Assay** | **Target** | **Primer name** | **Primer sequence** | **Probe name** | **Probe sequence** |
| --- | --- | --- | --- | --- | --- |
| SMB | eis | eis-F | 5’-CACAGGGTCACAGTCACAGAATC-3’ | eis-1 | 5’-Cy5-caggcggtcgtaatattcacgtgcacctggccgccgcctg-BHQ2-3’ |
|  |  | eis-R | 5’-GCATCGCGTGATCCTTTGCCAGAC-3’ | eis-2 | 5’-TxR-ctcgcggcatatgccacagtcggattctctgacgcgag-BHQ2-3’ |
|  | rrs | AMG-F | 5’-GCTAGTAATCGCAGATCAGCAACGCTGC-3’ | rrs-SMB | 5’-FAM-cacgaccgcccgtcacgtcatgaaagtcggtcgtg-BHQ1-3’ |
|  |  | AMG-R | 5’-CCTCCCGAGGGTTAGGCCACT-3’ |  |  |
|  | gyrA | gyrA-F | 5-CCGGTCGGTTGCCGAGACC-3 | QDR1 | 5-TxR-CCGTGCgcgcaccagggtgccctagatcgacacgtcGCACGG-BHQ2-3' |
|  |  | gyrA-R | 5-CCAGCGGGTAGCGCAGCGACCAG-3 | QDR2 | 5-Cy5-CCCGAGGgItgtcgtagattgacacgtcgccgcgcggCCTCGGG-BHQ2-3' |
|  | rpoB | rpoB-F2 | 5’-ACATCCGGCCGGTGGTCGCC-3’ | rpo1 | 5'-FAM-cgaccgCccatgaattggctcagctggctggtgAcggtcg-BHQ1-3' |
|  |  | rpoB-R | 5'-ACCTCCAGCCCGGCACGCTCACGT-3' | rpo2 | 5'-Cy5-ggcgcgaaccAcgacagcgggttgttctggtccatgaacgcgcc-BHQ2-3' |
|  |  |  |  | rpo3 | 5'-TxR-cgcgcgcaTcAccAacagtcggTgcttgtgggtcaacccgcgcg-BHQ2-3' |
| DLP | eis | eis-F2 | 5’-CGTGATCCTTTGCCAGACACT-3’ | eispP | 5′-FAM-ATCCGACTGTGGCATACGTGCACGTG-BHQ1-3′ |
|  |  | eis-R2 | 5’-TCGGTCGGGCTACACAGGGTCA-3’ |  |  |
|  | rrs | rrs-F2 | 5’-CGCGAGGTTAAGCGAATCC-3’ | rrsP | 5′-Cy5-TGTTACCGACTTTCATGACGTGACGGG-BHQ2-3′ |
|  |  | rrs-R2 | 5’-GGTACGGCTACCTTGTTACGACTT-3’ |  |  |
|  | gyrA | gyrA-F2 | 5’-CGGGCTTCGGTGTACCTCATC-3’ | gyraP | 5′-TxR-GTCGTAGATCGACGCGTCG-BHQ2-3′ |
|  |  | gyrA-R2 | 5’-TCGCCGGGTGCTCTATGCAAT-3’ |  |  |
|  | rpoB | rpobF | 5’-GCCGCGATCAAGGAGTTCTTC-3’ | rpobP1 | 5'-GGTTGTTCTGGTCCA(FAM)GAATTGGCTCAGC-BHQ1-3' |
|  |  | rpobR | 5’-CGGCACGCTCACGTGACAGAC-3’ | rpobP2 | 5'-GCCCCAGCGtCGACAG(FAM)CGGtGCTTGTGGG-BHQ1-3' |
