# Supplementary material for: Comparative Evaluation of Sloppy Molecular Beacon and Dual-Labeled Probe Melting Temperature Assays to Identify Mutations in Mycobacterium tuberculosis Resulting in Rifampin, Fluoroquinolone and Aminoglycoside Resistance
Source: PLoS One. 2015 May 4;10(5):e0126257. doi: 10.1371/journal.pone.0126257 (PMC4418795; doi:10.1371/journal.pone.0126257)
Supplement: S2 Table — (DOCX) [file pone.0126257.s005.docx]

**Table S2.**  Analytical specificity of the SMB and DLP assays for the different gene targets.

|  | *rpoB* Tm (°C) | | | | | *gyrA* Tm (°C) | | | *rrs* Tm (°C) | | *eis* Tm (°C) | | |
| --- | --- | --- | --- | --- | --- | --- | --- | --- | --- | --- | --- | --- | --- |
|  | rpo1 | rpo2 | rpo3 | rpobP1 | rpobP2 | QDR1 | QDR2 | gyraP | rrs | rrsP | eis1 | eis2 | eispP |
| H37Rv | 70.2 | 70.2 | 66.6 | 67.7 | 71.6 | 61.9 | 63.2 | 66.1 | 70.3 | 66.9 | 63.8 | 68.9 | 58.3 |
| *abscessus* | - | - | - | - | - | - | 56.8 | - | 70.2 | - | - | - | - |
| *scrofulaceum* | - | - | - | 55 | 64.3 | - | - | - | 70.2 | - | - | - | - |
| *celatum* | - | - | - | - | - | - | - | - | 70.1 | - | - | - | - |
| *haemophilum* | - | - | - | - | - | - | - | - | 70.3 | - | - | - | - |
| *asiaticum* | 66.9 | 69.4 | - | 64.6 | - | - | - | - | 70.2 | - | - | - | - |
| *kansasii* | 54.3 | 70.2 | - | 63.4 | - | - | - | - | 70.1 | - | - | - | - |
| *avium* | - | - | - | 54.9 | 64.2 | - | - | 53.8 | 70.2 | 67.9 | - | - | - |
| *flavescens* | - | - | - | - | - | - | - | - | 70.2 | - | - | - | - |
| *szulgai* | - | - | - | 64.5 | - | - | - | - | 70.2 | - | - | - | - |
| *terrae* | - | - | - | 64.5 | - | - | - | - | 70.1 | - | - | - | - |
| *fortuitum* | - | - | - | 50 | 61.6 | - | - | - | 70.2 | - | - | - | - |
| *intracellulare* | - | - | - | 55.2 | 64.4 | - | - | - | 70.2 | - | - | - | - |
| *marinum* | - | - | - | 60.5 | - | - | 57 | - | 70.2 | - | - | - | - |
| *xenopi* | - | - | - | - | - | - | - | - | - | - | - | - | - |
| *thermoresistibile* | - | - | - | - | - | - | - | - | 70.2 | - | - | - | - |
| *simiae* | - | - | - | 64.8 | - | - | - | - | 70.2 | - | - | - | - |
| *triviale* | - | 70.4 | 60.1 | 64.8 | - | - | - | - | 70.3 | - | - | - | - |
| *malmoense* | 57.3 | 72.7 | 62.2 | 65 | - | - | - | - | 70.4 | - | - | - | - |
| *smegmatis* | - | - | - | 54.9 | - | - | - | - | 70.3 | - | - | - | - |
